# The Intra-Meatal Application of Tadalafil Cream Versus Oral Administration Efficacy and Safety: Results from a Randomized, Two-Administration Route, Cross-Over Clinical Trial

**DOI:** 10.3390/jcm13216557

**Published:** 2024-10-31

**Authors:** Dragoș-Mihail Trifu, Daniel-Corneliu Leucuța, Martina-Luciana Pintea-Trifu, Florin Elec, Nicolae Crișan, Dan Eniu, Ioan Coman

**Affiliations:** 1 Department of Urology, Iuliu Hațieganu University of Medicine and Pharmacy, 400012 Cluj-Napoca, Romania; trifu.dragos.mihail@elearn.umfcluj.ro (D.-M.T.); florinelec@elearn.umfcluj.ro (F.E.); drnicolaecrisan@elearn.umfcluj.ro (N.C.); coman_ioan55@yahoo.com (I.C.); 2 Department of Urology, Municipal Blaj Hospital, 515400 Blaj, Romania; 3 Department of Urology, Endoplus Clinic, 400165 Cluj-Napoca, Romania; 4 Department of Medical Informatics and Biostatistics, Iuliu Hațieganu University of Medicine and Pharmacy, 400349 Cluj-Napoca, Romania; 5 Department of Cellular and Molecular Biology, Iuliu Hațieganu University of Medicine and Pharmacy, 400349 Cluj-Napoca, Romania; pintea.trifu.martina@elearn.umfcluj.ro; 6 Clinical Institute of Urology and Renal Transplantation, Iuliu Hațieganu University of Medicine and Pharmacy, 400000 Cluj-Napoca, Romania; 7 Department of Urology, Municipal Cluj Hospital, Iuliu Hațieganu University of Medicine and Pharmacy, 400139 Cluj-Napoca, Romania; 8 Department of Surgical Oncology, Iuliu Hațieganu University of Medicine and Pharmacy, 400000 Cluj-Napoca, Romania; daneniu@elearn.umfcluj.ro

**Keywords:** tadalafil, erectile disfunction, oral administration, local administration

## Abstract

**Background:** Tadalafil cream, a topically administered phosphodiesterase-5 inhibitor (PDE5), presents a potential alternative to oral PDE5 inhibitors like tadalafil for the treatment of erectile dysfunction (ED). This study evaluates the non-inferiority and potential superiority of tadalafil cream compared to oral tadalafil. **Methods:** This randomized controlled trial employed a cross-over design with two treatment periods of two weeks each, separated by a one-week washout phase. Thirty-five male participants aged 18–75 with diagnosed ED (International Index of Erectile Function–Erectile function: IIEF-EF score < 26) were randomized to receive either tadalafil cream or oral tadalafil. Tadalafil cream was applied topically, while tadalafil was taken orally. The primary endpoint was IIEF-EF, and secondary endpoints were measured using the International Index of Erectile Function (IIEF) domain scores. Adverse events and treatment preferences were also assessed. **Results:** Tadalafil cream showed a higher increase in sexual function across all IIEF domains compared to oral tadalafil. The lower bounds of the confidence interval [improvement: final−baseline scores between tadalafil cream and oral tadalafil 0.72 (95% CI −2.72–4.15)] were above the non-inferiority margin of −3.22, confirming tadalafil cream’s non-inferiority in the erectile function domain. In the intercourse satisfaction domain, tadalafil cream was superior to oral tadalafil. At the end of the trial, 88.57% of participants preferred tadalafil cream (95% CI 73.26%–96.79%), a result significantly above the non-inferiority margin that indicated superiority (*p* < 0.001). No systemic adverse events were reported for tadalafil cream, and significant differences in dizziness, headache, nasal congestion, and erythema were observed between the two treatments. **Conclusions:** Tadalafil cream is a safe and effective treatment for erectile dysfunction, demonstrating non-inferiority and potential superiority over oral tadalafil, with a high patient preference. Its topical administration offers a promising alternative for patients, particularly those with cardiovascular diseases where oral PDE5 inhibitors are contraindicated or less well tolerated.

## 1. Introduction

Erectile dysfunction (ED) is defined as a persistent or recurrent inability to obtain and/or maintain sufficient penile erection for satisfactory sexual intercourse [1]. The prevalence of ED is increased by aging, and in general, ED is less than 10% among males aged <40 years, less than 15% among males aged 40–49 years, 20%–30% among males aged 50–69 years, 20%–40% among males aged 60–69 years, and 50%–100% among males aged ≥70 years [2]. Etiological differentiation for ED consists of three major groups: (a) organic (vascular, neurogenic, hormonal, or anatomical causes), (b) psychogenic (stress, anxiety, or depression), and (c) mixt (involves both organic and psychogenic components, a combination frequently observed in many patients) [1].

The risk factors for ED may be classified into four categories, including (a) cardiovascular and metabolic disorders (such as diabetes mellitus, hypertension, hyperlipidemia, and obesity), (b) andrological or urological diseases (such as lower urinary tract symptoms), (c) psychosomatic and psychiatric disorders (such as depression, psychological stress, and antidepressants) and (d) lifestyle factors (such as smokers and sedentary lifestyle) [3,4,5]. Penile erection is a complex phenomenon that denotes a delicate, organized balance among vascular, neurological, and tissue compartments. It comprises penile arterial dilation and the smooth muscle relaxation of trabecular tissues with the stimulation of the mechanism of corporeal veno-occlusion [6,7,8,9]. NO plays a pivotal role in the improvement of endothelial function. NO enhances erectile function by increasing the endogenous amino acids that are required for synthesis and the ideal production of NO. Also, it affects nitric oxide–soluble guanylyl cyclase–protein kinase G signaling, which includes the stimulation of calcium-dependent potassium channels and blocks the up-regulated RhoA/Rho-kinase pathway [10]. It has been found that dietary supplementations with L-arginine could play an essential role in the treatment of ED [9]. According to the European Association of Urology (EAU) guidelines [11] for the management and treatment of ED, phosphodiesterase-5 inhibitor (PDE5i) constitutes the first line of oral therapy. PDE5i has been extensively studied in a wide population with different etiologies of ED, including men with renal failure, men with coronary artery diseases, and men after receiving a spinal cord injury (SCI). PDE5Is act by decelerating the degradation caused by the phosphodiesterase type 5 (PDE5) of cyclic guanosine monophosphate (cGMP), an essential regulator of intracellular calcium; this plays a pivotal role in the relaxation of smooth muscles and in the accumulation of blood in the corpora cavernosa that is needed for penile erection [12]. The efficacy of PDE5 inhibitors shows the significance of the nitric oxide–cGMP pathway through the inhibition of the degradation of the NO-generated cGMP. By selectively inhibiting the PDE-5, PDE-5 inhibitors thus conserve and maintain the NO-triggered enhancement in cGMP, which boosts the smooth muscle relaxation of cavernosal trabecular tissues [13,14].

Tadalafil is a selective and powerful PDEI5. It was approved for clinical use in 2003 after sildenafil and vardenafil approvals. It is characterized by a rapid onset (20 min) with a long duration of action (72 h) [15,16]. Its efficacy is not influenced by food intake [17].

According to an analysis of the World Health Organization (WHO) pharmacovigilance database, the most common systemic adverse reactions of PDE5i were poor drug efficacy (42.5%), headache (10.4% vs. 8.5%–27.6%), abnormal vision (8.4% vs. ≤4.6%), flushing (5.2% vs. 5.1%–16.5%), and dyspepsia (4.2% vs. 3.4%–11.1%). Priapism shows a significant correlation with tadalafil use (reporting odds ratio = 14.54; 95% confidence interval: 11.56–18.06). There is also a significantly higher reporting odds ratio for malignant melanoma (reporting odds ratio = 4.25; 95% confidence interval: 3.19–5.55) [18]. Nevertheless, we have found contrasting evidence in the literature referring to the topical side effects of the oral administration of PDE5i regarding melanoma [19]. There was also a case report of a patient presenting a rash caused by the oral administration of tadalafil that resolved following a low dose of topical corticosteroids [20].

This motivated the realization of this study with the aim to evaluate the non-inferiority and potential superiority of tadalafil cream, a topically administered PDE5 inhibitor, in comparison to oral tadalafil, an orally administered PDE5 inhibitor (tadalafil), for the treatment of erectile dysfunction in terms of avoiding systemic effects. To our knowledge, there are no studies that use the topical administration of PDE5i in erectile dysfunction. Specifically, this study seeks to assess improvements in erectile function and overall sexual satisfaction, as well as to document and compare the safety profiles and patient preferences for both treatments.

## 2. Materials and Methods

### 2.1. Trial Design

This cross-over randomized controlled trial (RCT) was designed to evaluate the non-inferiority and potential superiority of tadalafil cream, a locally administered drug, in comparison to tadalafil, an orally administered drug, for the treatment of erectile dysfunction (ED). The trial utilized a cross-over design featuring two distinct treatment periods of two weeks each. These periods were separated by a one-week washout phase to mitigate any carryover effects from the treatments. Each participant received both treatments in a randomized order. The trial was reported in compliance with the CONSORT 2010 statement for cross-over trials, ensuring a robust and transparent methodological framework. The trial was registered in the ISCRTN registry as ISRCTN80655184.

### 2.2. Participants

All participants were selected from “Prof. Dr. Ioan Puscas” City Hospital in Simleu Silvaniei, Romania, and from a private practice— the Regina Maria Clinic—during a urology examination that included an anamnesis, physical check-up, and ultrasound of the urogenital tract in search of abnormal findings. A consecutive sample was constituted. All patients were asked about their sex life, relationship status, sexual history, and sex duration. Also, they were interviewed about their psychosocial dynamics with their partner.

#### 2.2.1. Inclusion Criteria

Participants were recruited based on the following inclusion criteria: cisgender males aged 18–75 years; diagnosis of erectile dysfunction, confirmed by a score on the International Index of Erectile Function (IIEF)-EF < 26; willingness and ability to provide written informed consent, comply with the study procedures, and agree with the GDPR terms [21,22]; and general good health as determined by medical history and a physical examination. These criteria ensured participants could safely undergo both treatments.

#### 2.2.2. Exclusion Criteria

According to their medical history and current symptoms, patients were subjected to several blood tests, including complete blood count, testosterone level, prostatic specific antigen, sexually transmitted disease, follicle-stimulating hormone, luteinizing hormone, prolactin, and sex hormone-binding globulin tests. When the result indicated endocrine dysregulation, the patient was referred to an endocrinologist for further testing and treatment and excluded from the trial.

Participants were excluded based on the following criteria: the presence of severe cardiovascular, hepatic, renal, or hematological conditions that could interfere with the study treatments or outcomes; radiotherapy, pelvic surgery, or hormone therapy for prostate cancer; known hypersensitivity to any components of tadalafil cream or oral tadalafil; the use of other medications for erectile dysfunction during the study period to prevent potential drug interactions or confounding effects, such as beta-blockers and 5-α-reductase inhibitors; psychological or psychiatric disorders that could influence the study results or participant compliance; history of alcohol or drug abuse within the past year; or a PSA above 4 ng/mL.

### 2.3. Interventions

#### 2.3.1. Tadalafil Cream Administration

Tadalafil cream, the investigational product, was administered locally. Participants applied the gel 10–15 min before intercourse for a minimum of 3 times in 2 weeks according to the following protocol. Application instructions: participants were instructed to apply one dose of tadalafil cream to the penile meatus area. Monitoring: participants were asked to maintain a diary documenting the time of application and any local adverse reactions, such as irritation, redness, or itching, and any systemic side effects, such as headache, dizziness, or gastrointestinal disturbances in their diaries.

Tadalafil cream consists of Tadalafil 0.5 g + Ethoxydiglycol 0.3 g + liposomal Pentravan^®^ 2 g embedded in a urethral airless pen and packed in an insulated box. Pentravan^®^ is a transdermal carrier with a high degree of penetrability, which is applied to the gland, surrounding skin, and urethral meatus. It avoids hepatic passage and minimizes the adverse effects present during oral administration while also avoiding invasive treatments. Pentravan^®^ incorporates liposomal technology within its formulation to enhance the penetration of active ingredients through the skin. This design allows for effective encapsulation and deeper transdermal transport of medications, thereby improving their bioavailability and therapeutic efficacy. This transdermal cream is utilized extensively in applications such as hormone replacement therapy, pain management, and certain dermatological conditions. Its base consists of a non-greasy, oil-in-water emulsion that is both easy to apply and rapidly absorbed. This user-friendly aspect is crucial for patient adherence.

Additionally, the cream is engineered to reduce skin irritation and provides a consistent rate of drug delivery throughout the period of application. The pH and osmolarity of the formulation are carefully adjusted to match body tissues, optimizing both the comfort and safety of the drug delivery system for participants in the trial.

Each activation of the pen delivers a dose equivalent to a 20 mg Tadalafil tablet.

Tadalafil Cream Technical Data: The investigational product is a stiff, yellow gel with a slight lecithin odor. pH: The pH of the gel ranges from 4.0 to 5.5, making it suitable for topical application. Yield Value: The mechanical strength of the gel is quantified by a yield value ranging from 40 to 150 Pascal (Pa), indicating its resistance to flow under stress. Shelf Life: The product has a shelf life of 3 years.

#### 2.3.2. Oral Tadalafil Administration

Tadalafil, the comparator, was administered orally. Participants took the medication as follows. Dosage: 20 mg 30–60 min before a sexual encounter, with or without food. Adherence: participants recorded the time of intake and any systemic side effects such as headache, dizziness, or gastrointestinal disturbances in their diaries.

### 2.4. Outcomes

#### 2.4.1. Primary Outcome

The primary efficacy outcome was the International Index of Erectile Function (IIEF) Erectile Function domain score. The IIEF-EF is a validated questionnaire consisting of 6 questions (Q1, 2, 3, 4, 5, and 15) with scores ranging from 0 to 30 (each question has a score ranging from 0 to 6). Higher scores indicate better erectile function. The Erectile Function domain assesses explicitly the ability to achieve and maintain an erection that is sufficient for sexual intercourse. The scoring system categorizes the severity of erectile dysfunction as follows: scores of 1–10 indicate severe dysfunction, 11–16 indicate moderate dysfunction, 17–21 correspond to mild to moderate dysfunction, 22–25 represent mild dysfunction, and 26–30 indicate no dysfunction. The diagnosis of erectile dysfunction (ED) is a complex procedure that involves evaluating the patient’s medical history, conducting a physical examination, using standardized questionnaires, and considering laboratory tests to identify underlying causes.

#### 2.4.2. Secondary Outcomes

The secondary efficacy outcomes were the other IIEF domain scores: orgasmic function, sexual desire, intercourse satisfaction, and overall satisfaction. A secondary exploratory outcome was the treatment preference; the patients were asked at the end of the trial which of the two interventions they preferred. Adverse events were categorized as local (e.g., skin irritation and erythema) or systemic (e.g., headache and dizziness). These were documented throughout both study periods and assessed at each follow-up visit.

### 2.5. Sample Size

The sample size calculation was based on ensuring adequate power to detect non-inferiority within a −3.22 margin for the IIEF Erectile Function Domain score, as used by Kim et al. 2017 [23]. Assuming a standard deviation of 6.23 (based on the study of Ralph et al. 2018) [24] for the difference between IIEF scores and a significance level of 0.05, a total of 21 participants was deemed sufficient to achieve 95% power. We increased the number of participants to 35 since the funding for the study allowed it. The sample size calculation was performed with an online calculator [25] that used the formulas provided by Chow, Shao, and Wang [26].

### 2.6. Randomization and Blinding

#### 2.6.1. Randomization

Randomization was performed using a computer-generated randomization schedule with a 1:1 allocation ratio, with blocks of 4 and 6 [27]. Participants were randomly assigned to one of the two treatment sequences: sequence 1 (group A)—tadalafil cream for the first two-week period, followed by a week of washout period, and then oral tadalafil for the second two-week period, and sequence 2 (group B): oral tadalafil for the first two-week period, followed by a week of washout period, and then tadalafil cream for the second two-week period. The randomization list was accessible for a person not involved in the study. The participant was selected for the study after meeting the inclusion and exclusion criteria and signing the informed consent and the GDPR agreement [21,22]. Then, the principal investigator requested the intervention of the person possessing the randomization list without conveying any data about the patient. Thus, allocation concealment was enacted in this study.

#### 2.6.2. Blinding

Blinding of the participants was not feasible due to the different modes of administration (local gel vs. oral tablet). The participants completed the questionnaires on their own; thus, the outcome assessors were the participants.

### 2.7. Washout Period

A one-week washout period was implemented between the two treatment periods. This duration was chosen based on the pharmacokinetic profiles of both tadalafil cream and oral tadalafil, ensuring that any residual effects of the first treatment would not influence the outcomes of the second treatment. During the washout period, participants were instructed to refrain from using any erectile dysfunction medications.

### 2.8. Data Collection

Data were collected at four key time points: (1) initial measurement period 1: baseline data were collected before starting the first treatment, including demographic information, medical history, and baseline IIEF scores; (2) final measurement period 1: at the end of the first two-week treatment period, participants completed the IIEF questionnaire and reported any adverse events; (3) initial measurement period 2: IIEF scores after the one-week washout period and before starting the second treatment were collected; (4) final measurement period 2: at the end of the second two-week treatment period, the final IIEF scores, adverse event reports, and intervention preference were collected.

### 2.9. Statistical Analysis

Categorical data were presented as counts and percentages. Continuous data were presented as means and standard deviations, and, in the case of non-normality, as medians and interquartile ranges. To assess the intervention efficacy, linear mixed models predicting changes (final−initial) in the international index scores of the erectile function domains were used depending on the intervention, after adjusting for the period, while considering the interaction between the treatment and period, and while considering random effects for patients. The results of the model were presented by the least squares coefficients with a 95% confidence interval (CI) and *p*-values. The *p*-value of interest for the endpoints corresponded to the intervention. The primary analysis aimed to establish the non-inferiority of tadalafil cream compared to oral tadalafil for the IIEF Erectile Function Domain score. Non-inferiority was defined as the lower bound of the 95% confidence interval for a difference in mean scores (tadalafil cream—oral tadalafil) greater than −3.22 [23]. If non-inferiority was established, a subsequent superiority analysis was conducted to determine if tadalafil cream was superior to oral tadalafil. The confidence interval from the linear mixed model should be above 0 to establish superiority. Concerning the preference for one of the two interventions assessed at the end of the trial, a binomial exact test with a 95% CI was computed. The non-inferiority limit was set for tadalafil cream to be not more than 10% worse than oral tadalafil, thus above 40%. The superiority limit was considered a confidence interval lower than 50% [28]. For adverse reactions, an exact Mc Nemar test was used. For all statistical tests, a 0.05 level of significance was used. All analyses were carried out with R environment for statistical computing and graphics (R Foundation for Statistical Computing, Vienna, Austria), version 4.2.3 [29].

### 2.10. Data Handling

A per-protocol analysis was pursued to evaluate the primary outcome, although since all participants followed the protocol, and none were lost to follow-up, the analysis was equivalent to the intention-to-treat one. No data were missing, so no multiple imputations were needed to address this issue.

### 2.11. Ethical Considerations

The study was conducted in accordance with the principles outlined in the Declaration of Helsinki and was approved by the Ethics Committee of the Iuliu Hațieganu University of Medicine and Pharmacy, Cluj-Napoca (AVZ 14/3 February 2022). All participants provided written informed consent before enrollment, ensuring their voluntary participation and understanding of the study procedures. Participants were assured of their right to withdraw from the study at any time without penalty.

## 3. Results

**Figure 1:**
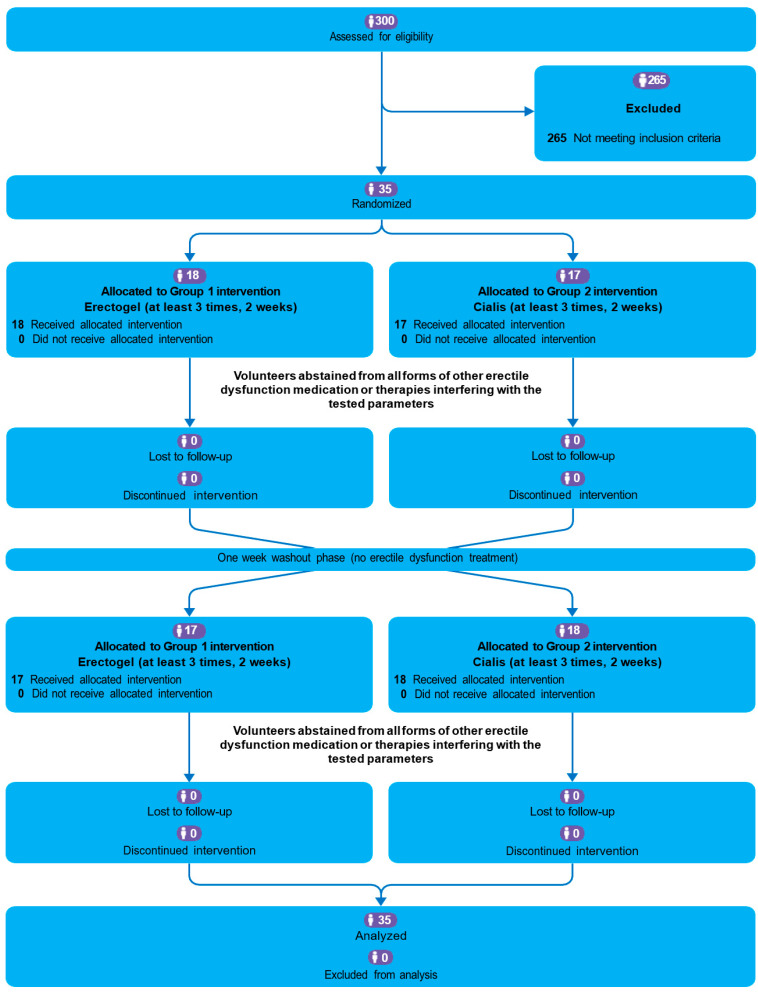
CONSORT flow diagram for the cross-over trial.

Out of 300 participants assessed for eligibility, 35 were included in the trial (Figure 1). In the first period, 18 participants received tadalafil cream and 17 received oral tadalafil. After the washout period, each participant switched interventions. No patient was lost to the follow-up, and all participants followed the protocol.

The baseline characteristics of patients are presented in Table 1. The mean age was 49 years (13.5 standard deviations), ranging from 21 to 69 years. The most frequent etiology of erectile dysfunction (ED) was of mixed origin (has an organic cause as well as psychogenic factors), accounting for 57.1% of cases. (20). The median ED duration was 12 (IQR 8.5–15.5) months. Prostate pathology (chronic prostatitis and bladder outlet obstruction) was present in 51.4% (18) of the patients. The most frequent comorbidity was hypertension, 42.9% (15). There were 34.3% (12) smokers and 62.9% (22) who consumed alcohol.

The baseline and final IIEF scores for each period are presented in Table 2. The initial baseline IIEF scores for the first period were higher in the oral tadalafil receiving group compared to the tadalafil cream group. The most important differences were erectile function and overall satisfaction. At the baseline of the second period, this situation was inversed. The tadalafil cream had higher IIEF scores compared to the oral tadalafil receiving group, the differences being more pronounced compared to those in the first period. The most important differences were erectile function and overall satisfaction.

When looking at all subjects at the baseline by the intervention received, irrespective of the period, the tadalafil cream-receiving participants had higher scores compared to those receiving oral tadalafil (Table 3). Nevertheless, the differences were very small (under 0.5 points).

The observed IIEF scores increased more in the first period for the tadalafil cream users compared to the oral tadalafil users (Table 4). In the second period, the IIEF scores increased more for the tadalafil cream users concerning erectile function and intercourse satisfaction and for oral tadalafil users concerning sexual desire, orgasmic function, and overall satisfaction.

When looking at all the participants, there were higher observed increases in erectile function, orgasmic function, and intercourse satisfaction during the tadalafil cream intervention, while sexual desire and overall satisfaction had higher observed increases during oral tadalafil intervention (Table 5) in the univariate analysis.

### 3.1. Main Outcome Analyses

The main analysis of the effects of the interventions was performed using a linear mixed model predicting changes in IIEF scores (final−baseline) that was adjusted for the period and included interactions and random effects for patients (Table 6). On average, for all the IIEF domains, the increase in sexual functions was higher in the tadalafil cream users compared to the oral tadalafil users. All confidence intervals’ lower bounds were above the non-inferiority margin of −3.22, indicating that tadalafil cream was non-inferior compared to oral tadalafil for all IIEF domains, including the main endpoint of erectile function. Furthermore, for the intercourse satisfaction domain, tadalafil cream was superior to oral tadalafil. There was a period effect concerning sexual desire, the increase in it being higher in the second period compared to the first period.

### 3.2. Preference for a Specific Intervention

The percentage of preference of the participants at the end of the trial for tadalafil cream was 88.57% (95% CI 73.26%–96.79%). The result is above the non-inferiority margin of 40%. Furthermore, the result shows superiority, being statistically significant, with a *p*-value of <0.001.

### 3.3. Adverse Reactions

There were no systemic adverse events related to tadalafil cream usage. There were no local adverse reactions to oral tadalafil. There were significant differences (Table 7) concerning dizziness, cephalea, nasal congestion, and erythema.

### 3.4. Sincerity and Embarrassment

Finally, the participants in the trial were asked about their sincerity regarding the questions they were asked during the trial (Table 8). In both periods, the percentage of being completely sincere was above 97%. There was one participant (2.86%) affirming being mostly sincere in the second period, and one participant (2.86%) affirming being sincere to some extent in the first period. No patient claimed to be completely insincere. About 30% of the participants felt a little embarrassed while answering the questions, while 14% in the first period, followed by 8% in the second period, felt moderately embarrassed. The sensation was less intense in the second period compared to the first one.

## 4. Discussion

The results of our cross-over randomized controlled trial (RCT) provide compelling evidence that tadalafil cream was non-inferior to oral tadalafil concerning the erectile function domain. Utilizing a linear mixed model to predict changes in IIEF scores, our analysis showed that tadalafil cream led to improvements across all domains of sexual function compared to oral tadalafil but offered statistically significant superiority benefits concerning the intercourse satisfaction domain. Additionally, a notable period effect was observed in sexual desire, with higher increases reported in the second period. Participant preference overwhelmingly favored tadalafil cream, with 88.57% expressing a preference for it over oral tadalafil, a result that significantly surpassed the non-inferiority margin and underscored its superior acceptability. Safety profiles were also favorable, with no systemic adverse events reported for tadalafil cream and only minor local adverse reactions observed with oral tadalafil. The high degree of sincerity reported by participants, coupled with a reduction in embarrassment over time, further supported the reliability of our findings. These results suggested that tadalafil cream could be a preferred alternative for individuals seeking effective and well-tolerated treatments for erectile dysfunction.

There are few studies regarding the topical use of PDE5i in the literature. Searching Pubmed using the terms “topical” and “tadalafil” between the years 2003 and 2024 returned about two studies speaking of the topical use for PDE5i, especially tadalafil. One of them demonstrated the use of tadalafil for wound healing by activating vasodilatation [30]. The other one demonstrated the superiority of topical tadalafil skin permeability vs. sildenafil and vardenafil in a rodent model and also non-inferiority to oral use [31]. There are no RCTs regarding the topical use of PDE5i in humans. The only data in the literature regarding topical treatment for ED are for prostaglandin E1 (alprostadil) and hormones (testosterone) with the Pentravan^®^ delivery system [32,33,34,35].

Although limited, the available data suggest promising outcomes regarding the topical treatment of erectile dysfunction [36]. Significant technological advancements are being made in the development of transdermal delivery systems for various molecules used in the treatment of endocrine and andrological conditions. These systems have the potential to achieve efficacy at least equivalent to oral administration while also circumventing a range of adverse effects that make these treatments prohibitive for certain patient groups. In this context, there is a critical need to develop clinical trials focusing on the topical treatment of erectile dysfunction and related conditions.

### 4.1. Limitations

This study has several limitations that warrant consideration. The lack of blinding due to the distinct modes of administration (topical gel vs. oral tablet) may have introduced bias, as participants were aware of which treatment they were receiving, potentially influencing their reported outcomes. The study sample size, though calculated to ensure adequate power, remained relatively small, which could have limited the generalizability of the findings. The reliance on self-reported measures for both efficacy and adverse events could introduce reporting bias, as participants could underreport or overreport their experiences based on their subjectivity.

### 4.2. Strengths

Despite these limitations, the study has several notable strengths that enhance the validity and reliability of its findings. The main strength of the study is that this is one of the few studies using the topical administration of PDE5i in erectile dysfunction. The use of a cross-over design is a significant strength, as it allows each participant to serve as their own control, thereby reducing inter-subject variability and increasing the statistical power of the study. Additionally, the high level of participant adherence and the absence of dropouts strengthen the internal validity of the study. The detailed monitoring of adverse events and the inclusion of both local and systemic side effects provide a comprehensive safety profile for both treatments. Finally, the high reported sincerity of participants in answering the questionnaires adds to the credibility of the self-reported outcomes, suggesting that the data accurately reflect the participants’ experiences.

### 4.3. Clinical Utility

This study demonstrates the clinical utility of tadalafil cream as a viable and effective treatment option for erectile dysfunction, offering advantages over oral tadalafil in terms of improved sexual function and higher patient preference. The topical administration of tadalafil cream provides a non-invasive alternative that is associated with fewer systemic side effects, making it particularly suitable for patients who may have relative contraindications to oral medications (history of myocardial infarction, stroke, or life-threatening arrhythmia in the past six months; resting hypotension or hypertension; history of heart failure or unstable angina; and concomitant administration of alpha-blockers). The findings suggest that tadalafil cream can be confidently recommended as a first-line treatment for erectile dysfunction, potentially enhancing patient satisfaction and adherence to therapy.

### 4.4. Future Directions

Future research should focus on expanding the study population to include a more diverse demographic to enhance the generalizability of the findings. Additionally, exploring the long-term efficacy and safety of tadalafil cream through extended follow-up periods will provide valuable insights into its sustained benefits and potential risks.

## 5. Conclusions

In conclusion, this cross-over randomized controlled trial provides compelling evidence that tadalafil cream, a topically administered PDE5 inhibitor, is a viable and effective treatment for erectile dysfunction. Our findings indicate that tadalafil cream is non-inferior to oral tadalafil in all domains of sexual function, with superiority in intercourse satisfaction and high patient preference. The safety profile of tadalafil cream is favorable, with no systemic side effects compared to oral tadalafil. These results suggest that tadalafil cream offers a promising alternative for individuals seeking effective and well-tolerated treatments for erectile dysfunction. Given its favorable safety profile and topical administration, tadalafil cream may be particularly beneficial for patients with cardiovascular diseases who cannot use or have difficulty tolerating oral PDE5 inhibitors. Further research is warranted to confirm these findings in larger and more diverse populations, as well as to explore the long-term efficacy and safety of tadalafil cream.

## Figures and Tables

**Table 1:** Baseline characteristics of patients.

Characteristic	Number (%)
	(n = 35)
Age (years), average age (DS):	49.06 (13.46)
Duration ED (months), median (IQR):	12 (8.5–15.5)
Etiology	
Psychogenic:	15/35 (42.86)
Mixed:	20/35 (57.14)
Prostate pathology	18/35 (51.43)
Prostate pathology treatment	13/35 (37.14)
Sexual orientation	
Heterosexual:	33/35 (94.29)
Homosexual:	1/35 (2.86)
Other:	1/35 (2.86)
Marital status	
Married:	23/35 (65.71)
Divorced:	1/35 (2.86)
Unmarried:	8/35 (22.86)
Widowed:	3/35 (8.57)
LUTS	
Genital pruritus:	1/35 (2.86)
Urethral discharge:	0/35 (0)
Odynorgasmia:	1/35 (2.86)
Dysuria:	4/35 (11.43)
Haematospermia:	0/35 (0)
Hematuria:	0/35 (0)
Smarting ejaculation:	0/35 (0)
Smarting micturition:	5/35 (14.29)
Comorbidities	
Ischemic heart disease:	1/35 (2.86)
Diabetes mellitus:	3/35 (8.57)
Arterial hypertension:	15/35 (42.86)
Obesity:	2/35 (5.71)
Vertebral/pelvic trauma:	1/35 (2.86)
Toxic substance use	
Alcohol	
Abstinent:	13/35 (37.14)
Low:	7/35 (20)
Moderate:	9/35 (25.72)
Heavy:	6/35 (17.14)
Smoker:	12/35 (34.29)
No cigars (21–30/day vs. 5–10/day):	8/12 (66.67)

**Table 2:** International index of erectile function at the beginning and the end of each study period.

	Period 1				Period 2			
	Baseline		Final		Baseline		Final	
International Index of Erectile Function	Tadalafil Cream	Oral Tadalafil	Tadalafil Cream	Oral Tadalafil	Tadalafil Cream	Oral Tadalafil	Tadalafil Cream	Oral Tadalafil
	(n = 18)	(n = 17)	(n = 18)	(n = 17)	(n = 17)	(n = 18)	(n = 17)	(n = 18)
Erectile function	15	17.29	19.83	21.41	19.41	16.11	24.53	20.72
	(6.55)	(7.12)	(6.6)	(5.52)	(5.9)	(6.99)	(4.21)	(6.76)
Sexual desire	6.83	7.76	7.33	7.82	7.76	6.11	8.12	7.11
	(2.12)	(1.35)	(1.94)	(1.24)	(1.35)	(2.14)	(1.05)	(1.53)
Orgasmic function	6.83	7	8.44	7.88	7.47	6.83	8.76	8.44
	(3.09)	(3.14)	(1.79)	(2.69)	(2.55)	(3.43)	(1.6)	(2.09)
Intercourse satisfaction	7.17	8.18	10.39	9.65	9.18	8.06	11	9.67
	(3.55)	(3.86)	(2.7)	(2.74)	(2.92)	(3.44)	(2.29)	(3.22)
Overall satisfaction	4.56	6.12	5.94	6.94	7.06	4.94	7.88	6.44
	(1.82)	(2.8)	(1.98)	(2.59)	(2.66)	(1.73)	(1.73)	(1.5)

Values are presented as means and standard deviations.

**Table 3:** Baseline and final values for the international index of erectile function for all participants by intervention.

	Baseline		Final	
International Index of Erectile Function	Oral Tadalafil	Tadalafil Cream	Oral Tadalafil	Tadalafil Cream
	(n = 35)	(n = 35)	(n = 35)	(n = 35)
Erectile function	16.69	17.14	21.06	22.11
	(6.98)	(6.54)	(6.11)	(5.98)
Sexual desire	6.91	7.29	7.46	7.71
	(1.96)	(1.82)	(1.42)	(1.6)
Orgasmic function	6.91	7.14	8.17	8.6
	(3.25)	(2.82)	(2.38)	(1.68)
Intercourse satisfaction	8.11	8.14	9.66	10.69
	(3.6)	(3.37)	(2.95)	(2.49)
Overall satisfaction	5.51	5.77	6.69	6.89
	(2.36)	(2.57)	(2.08)	(2.08)

Values are presented as means and standard deviations.

**Table 4:** International index of erectile function change between final and baseline values by study period.

	First Period		Second Period	
International Index of Erectile Function	Tadalafil Cream	Oral Tadalafil	Tadalafil Cream	Oral Tadalafil
	(n = 18)	(n = 17)	(n = 17)	(n = 18)
Erectile function	4.83 (7.71)	4.12 (4.17)	5.12 (3.12)	4.61 (3.93)
Sexual desire	0.5 (1.34)	0.06 (0.56)	0.35 (0.7)	1 (1.08)
Orgasmic function	1.61 (1.94)	0.88 (1.93)	1.29 (1.49)	1.61 (2.66)
Intercourse satisfaction	3.22 (3.37)	1.47 (1.55)	1.82 (1.74)	1.61 (1.54)
Overall satisfaction	1.39 (1.75)	0.82 (1.38)	0.82 (1.29)	1.5 (1.2)

Values are presented as means and standard deviations.

**Table 5:** International index of erectile function change between final and baseline values on all subjects.

International Index of Erectile Function	Tadalafil Cream	Oral Tadalafil	Difference (95% CI)
	(n = 35)	(n = 35)	
Erectile function	4.97 (5.86)	4.37 (3.99)	0.6 (−0.97–2.17)
Sexual desire	0.43 (1.07)	0.54 (0.98)	−0.11 (−0.51–0.28)
Orgasmic function	1.46 (1.72)	1.26 (2.33)	0.2 (−0.61–1.01)
Intercourse satisfaction	2.54 (2.76)	1.54 (1.52)	1 (0.27–1.73)
Overall satisfaction	1.11 (1.55)	1.17 (1.32)	−0.06 (−0.6–0.49)

Values are presented as means and standard deviations. CI is the confidence interval for paired differences.

**Table 6:** Linear mixed models predicting change (final−initial) scores of the international index of erectile function domains in the function of the treatment adjusted for the period with interactions and random effects for patients.

Dependent Variable	Intervention (95% CI)	*p*	Period (95% CI)	*p*	Intervention * Period Interaction (95% CI)	*p*
Erectile function	0.72	0.679	0.49	0.775	−0.21	0.946
	(−2.72–4.15)		(−2.94–3.93)		(−6.33–5.91)	
Sexual desire	0.44	0.188	0.94	0.006	−1.09	0.051
	(−0.22–1.1)		(0.28–1.6)		(−2.18–0.01)	
Orgasmic function	0.73	0.299	0.73	0.299	−1.05	0.362
	(−0.66–2.12)		(−0.66–2.12)		(−3.32–1.23)	
Intercourse satisfaction	1.75	0.022	0.14	0.851	−1.54	0.247
	(0.26–3.24)		(−1.35–1.63)		(−4.17–1.09)	
Overall satisfaction	0.57	0.245	0.68	0.165	−1.24	0.123
	(−0.4–1.53)		(−0.29–1.64)		(−2.83–0.34)	

Intervention: tadalafil cream vs. oral tadalafil. Period coded as 0 for the first one and 1 for the second one. CI, confidence interval.

**Table 7:** Adverse reaction comparison between tadalafil cream and oral tadalafil.

Treatment	Oral Tadalafil	Tadalafil Cream	*p*-Value
	(n = 35)	(n = 35)	
Systemic reactions			
Dizziness, n (%)	9 (25.71)	0 (0)	0.004
Cephalea, n (%)	6 (17.14)	0 (0)	0.031
Nazal congestion, n (%)	12 (34.29)	0 (0)	<0.001
Dyspepsia, n (%)	4 (11.43)	0 (0)	0.125
Facial redness, n (%)	2 (5.71)	0 (0)	0.5
Lipotomy, n (%)	0 (0)	0 (0)	1
Cyanopsia, n (%)	1 (2.86)	0 (0)	1
Local reactions			
Burn sensation, n (%)	0 (0)	3 (8.57)	0.25
Pain, n (%)	0 (0)	1 (2.86)	1
Erythema, n (%)	0 (0)	6 (17.14)	0.031
Itchiness	0 (0)	3 (8.57)	0.25
Smarting	0 (0)	1 (2.86)	1

**Table 8:** How sincere and embarrassed were the participants regarding the questions they were asked during the trial?

Question	Period 1	Period 2
	(n = 35)	(n = 35)
How sincere were your answers, n (%)		
Entirely	34 (97.14)	34 (97.14)
Mostly	0 (0)	1 (2.86)
To some extent	1 (2.86)	0 (0)
Not at all	0 (0)	0 (0)
How embarrassed you felt when answering the questions, n (%)		
Not at all	18 (51.43)	20 (57.14)
A little	11 (31.43)	12 (34.29)
Moderately	5 (14.29)	3 (8.57)
Very	1 (2.86)	0 (0)

## Data Availability

The dataset is available upon request from the authors.
